# Suggestion-Induced Modulation of Semantic Priming during Functional Magnetic Resonance Imaging

**DOI:** 10.1371/journal.pone.0123686

**Published:** 2015-04-29

**Authors:** Martin Ulrich, Markus Kiefer, Walter Bongartz, Georg Grön, Klaus Hoenig

**Affiliations:** 1 Department of Psychiatry, University of Ulm, Ulm, Germany; 2 Klingenberg Institute for Clinical Hypnosis, Konstanz, Germany; 3 Department of Psychosomatic Medicine and Psychotherapy, University of Ulm, Ulm, Germany; Brown University, UNITED STATES

## Abstract

Using functional magnetic resonance imaging during a primed visual lexical decision task, we investigated the neural and functional mechanisms underlying modulations of semantic word processing through hypnotic suggestions aimed at altering lexical processing of primes. The priming task was to discriminate between target words and pseudowords presented 200 ms after the prime word which was semantically related or unrelated to the target. In a counterbalanced study design, each participant performed the task once at normal wakefulness and once after the administration of hypnotic suggestions to perceive the prime as a meaningless symbol of a foreign language. Neural correlates of priming were defined as significantly lower activations upon semantically related compared to unrelated trials. We found significant suggestive treatment-induced reductions in neural priming, albeit irrespective of the degree of suggestibility. Neural priming was attenuated upon suggestive treatment compared with normal wakefulness in brain regions supporting automatic (fusiform gyrus) and controlled semantic processing (superior and middle temporal gyri, pre- and postcentral gyri, and supplementary motor area). Hence, suggestions reduced semantic word processing by conjointly dampening both automatic and strategic semantic processes.

## Introduction

According to classical theories, higher-level top-down influences like attentional control or task set configurations are believed to be restricted to conscious processing. Automatic processes, in turn, are conceived to occur autonomously, often unconsciously and without top-down modulation [1, 2]. However, early refined models of automaticity suggest that the occurrence of automatic processes might also rely on specific configurations of the cognitive system [3]. In extension of these suggestions Kiefer and colleagues developed the attentional sensitization model of unconscious cognition and have recently shown that the occurrence of a certain automatic process depends on prior sensitization of the cognitive system for instance by specific task sets (e.g., orientation to semantic word features vs. perceptual word features) [4–7]. Behavioral, electrophysiological, and neuroimaging evidence supports that unconscious automatic processes can be modulated by top-down factors (see [8], for an overview).

The Stroop color word interference effect [9] is just one well-known example for the occurrence of unconscious automatic processes. Central to the Stroop interference is the automaticity of reading (e.g., [10, 11]) which leads to prolonged response latencies when the task-irrelevant color word does not match the to-be-named ink color in which this word is written. However, even for this effect, strict automaticity in the sense of the classical criteria of automaticity [1, 2] has recently been questioned by reports about modulation of the Stroop effect through post-hypnotic suggestions (e.g., [12–15]). This finding is in line with the attentional sensitization model [6] and supports the idea that hypnotic suggestion exerts its influence through modulating attentional control of unconscious automatic processes [16].

Within the context of language processing, in addition to the Stroop effect, semantic priming is also thought to probe automatic aspects of word processing. Semantic priming is typically investigated by use of a primed lexical decision task (e.g., [17]) where subjects are to discriminate between target words and pseudowords. Preceding the target a prime word is presented which can be semantically related (R) or unrelated (U) to the target stimulus (in case the target is a word). Usually, decisions on semantically related target words are faster and less error prone compared to unrelated target words, a phenomenon called “semantic priming” (see [18], for a review). Mechanisms that have been proposed to explain semantic priming can be divided into those involving automatic processes most likely reflecting pre-activation of distributed semantic features [19–23], and strategic processes such as expectancy generation [1, 24] and semantic matching [24–26]. Previous work within the context of the attentional sensitization model of unconscious cognition [6] has shown that attentional orientation towards word meaning can modulate automatic semantic priming [4–7]. Comparing masked with unmasked primes, we recently reported data supportive of the notion that the automatic aspects of semantic priming elicited by unconsciously perceived masked primes most likely involve a portion of the left fusiform gyrus (FFG) while the bilateral superior temporal gyri (STG), the pre- and postcentral gyri (PrG, PoG), the supplementary motor area proper (SMA proper), and the superior parietal lobules (SPL) were most likely involved in strategic processes tied to supraliminal priming, such as semantic matching [27].

In the present study, we attempted to modulate semantic priming effects using hypnotic suggestions similar to the studies conducted by Raz and colleagues [12, 15, 28] and with the principal idea that in the context of a lexical decision task words can be “stripped of their meaning by suggestion, yet still be responded to as words” [16]. In a typical semantic priming design, this would imply that the subject is unable to access the meaning of the prime word while being able to perform a lexical decision upon an immediately following target item. Suggestions were applied after participants had undergone a hypnotic induction procedure. All subjects performed the task also at normal wakefulness without suggestions. To gain insight into the neural and functional processes involved in a possible modulation by hypnotic suggestion, brain activity during semantic priming was measured using functional magnetic resonance imaging (fMRI).

We hypothesized that semantic priming may be attenuated by hypnotic suggestion relative to normal wakefulness by modulating the attentional set of processing the prime word. Under normal wakefulness without specific prime-related task instruction, participants typically read the prime words and access their meaning. In contrast, when receiving a hypnotic suggestion to perceive the prime words as meaningless symbols, this might induce an attentional set that leads to a desensitization of semantic pathways. As described above, semantic priming with visible primes is typically based on both automatic and controlled semantic processes. Hypnotic suggestion might modulate semantic priming effects through desensitizing either semantic processing pathway. Our previous results [27] indicated that automatic semantic priming processes and strategic priming processes may involve distinct brain regions. Suggestive modulation of automatic processes of semantic priming should therefore attenuate priming within the FFG. Conversely, if the hypnotic-suggestive treatment had an effect on strategic processes, priming should be attenuated in the STG, the PrG/PoG, the SMA proper, or the SPL. We also included a standard measure of suggestibility to test whether this trait-like capacity [29] might be related to a possible modulation of semantic priming. In the context of the Stroop task, the reduction of the automaticity of reading has been observed to be restricted to subjects with high imaginative suggestibility [15], while another study reported substantial modulation of the Stroop effect also in subjects with low suggestibility [13]. Thus, we hypothesized that the hypnotic suggestion should have more impact on high- than on low-suggestible individuals. The high-suggestible group should thus show diminished semantic priming in comparison to participants of low suggestibility.

## Materials and Methods

### Participants

Participants were the same as those reported in our previous masking study [27]. Twenty-four right-handed German native speakers (mean age = 22.3 years, standard deviation (SD) = 1.7 years) were recruited from a pool of university students (n = 150) that had undergone a screening for hypnotic susceptibility using a German version [30] of the Harvard Group Scale of Hypnotic Susceptibility, Form A (HGSHS:A) [29]. The HGSHS:A expresses individual levels of suggestibility in a sum score from 1 to 11. Fourteen participants (eight female) were of higher suggestibility (HS), with a mean HGSHS:A score of 9.6 (SD = 0.8). A second group comprised ten subjects (six female) of lower suggestibility (LS; mean HGSHS:A score = 3.5, SD = 1.5). Neither age nor handedness (assessed by the Edinburgh Inventory [31]) differed significantly between groups (mean age: HS: 21.7, LS: 23.0; t(22) = 1.91, p = 0.069; mean right-handedness quotient in %: HS: 79.7, LS: 89.3; t(22) = 1.55, p = 0.135). There were no reports of psychiatric/neurological disorders or any other contraindications regarding the fMRI procedure. Written informed consent was obtained prior to the experiment. The study has been approved by the local ethics committee at the University of Ulm and was in accordance with the Declaration of Helsinki.

### Stimuli

Stimulus material consisted of German words (nouns, verbs, and adjectives) and pronounceable pseudowords, arranged in parallel lists comprising 60 word-word and 60 word-pseudoword (P) pairs each, derived from earlier priming studies (e.g., [32]). Half of the word-word pairs consisted of words with a related meaning (R) while the words of the other half were unrelated in meaning (U). The first item of each pair served as prime and the second item as target. Primes and targets were matched for average word length and frequency [33] across and within lists. Two further sets of (unmatched) stimuli were created for use as practice runs (10 R, 10 U, and 20 P pairs each). There were no stimulus repetitions within or across lists.

After obtaining a pseudo-randomized trial sequence using “Optseq2” (http://surfer.nmr.mgh.harvard.edu/optseq/; see also [34]), onsets were jittered by randomly adding fractions of the fMRI repetition time in order to achieve finer-grained temporal sampling of the hemodynamic response. Average trial onset asynchronies were 24.0 s (R), 23.8 s (U), and 12.3 s (P). Experimental Run Time System 3.35 (BeriSoft Cooperation, Frankfurt/ Main, Germany) was used for visual stimulation and recording of participants’ reactions. Stimuli appeared in white 24 point IBM8BIT font centered on a black background. Visual angle was 1.0° vertically and, depending on word length, 1.4° - 5.7° horizontally. Stimuli were delivered at a resolution of 800 x 600 pixels and a screen refresh frequency of 60 Hz through MRI compatible video goggles (VisuaStim Digital, Resonance Technology Inc., Northridge, California, USA).

### Procedure

The task was a primed visual lexical decision task [17]. First, a prime was presented with 200 ms duration. Directly afterwards a target was shown for 500 ms. Participants were asked to press a button with their right index finger whenever the target was a German word, and to press a different button with their right middle finger when the target was a pseudoword. A time window of 2000 ms beginning with target onset was provided to perform the decision. During that period, after target-offset, a black screen was shown for 1500 ms. Instructions stressed a balance between response speed and accuracy. During the variable intertrial interval (mean = 3.9 s, maximum = 25.4 s) subjects were asked to fixate a crosshair centered on the screen. Further phases of fixation were placed at the beginning (13.2 s) and at the end (22 s) of the fMRI session to account for scanner equilibration and to capture the hemodynamic response associated with the last trial, respectively. Each participant performed the task once under hypnotic suggestion (= treatment, “Treat”) and once under normal wakefulness (= no treatment, “noT”), with the order of conditions counterbalanced across participants. Depending on session order, the gap in time between both sessions was roughly 15 to 30 minutes. Notably, for a given participant prime and target stimuli did not repeat because different matched stimulus lists were assigned to the experimental sessions in a counterbalanced fashion.

The treatment session began with the induction of a state of trance following the procedure by Weitzenhoffer and Hilgard [35], translated into German and adjusted to the requirements of an MRI environment. The induction was performed live and verbally conveyed to participants using the headset equipment of the MR scanner. First, subjects were ensured that no harm would happen to them by any means. Then they were asked to direct their attention to a white fixation cross that was placed in the upper third of an otherwise black screen. By administering appropriate suggestions, participants should get increasingly tired until their eyes were finally closed. In parallel, suggestions were given to make subjects relaxed, calmed, and detached. The noise of the scanner was suggested as being a “background protecting from and shielding against disturbance” to prevent trance de-induction (upon scanner onset). Following the induction phase, task-specific instructions were administered in order to dampen down semantic processing of the prime without affecting target processing or lexical decision. The suggestions followed the ones used by Raz and colleagues [12, 15, 28], modified in certain respects to conform to the present experimental design. In contrast to the studies by Raz et al. where suggestions took effect in a posthypnotic context, present suggestions were given in a hypnotic context. The suggestions read as follows:

“Very soon you will be performing the computer task. There will always appear two character strings in succession in the middle of the screen. Just indicate by button press at any time whether or not the second string is a German word. As you already know, you will not have to care about the first string of each pair. You will perceive each first string as gibberish of characters. The first string of each pair will feel like a symbol of a foreign language that you do not know, and you will not attempt to attribute any meaning to them. There would not be any point in that anyhow because you know how difficult … how impossible it is to comprehend the meaning of entirely foreign symbols … like these first strings. Nevertheless you will be able to easily fixate both strings comparably well and to see them clearly and sharply. You will find that you can perform the task easily and effortlessly.”

After the suggestions, participants were prompted to open their eyes, and a very brief and final instruction was given which buttons were to press for the lexical decision. Thereafter, the MR scanning started. Upon task completion, the suggestive treatment session ended with a de-induction of the state of trance by verbally counting backwards from 10. When participants heard the number “1” they were to feel “wide wake, rested, and relaxed”. Participants performed the task also at normal wakefulness (noT), without preceding trance induction or application of any suggestions. Prior to both experimental sessions (noT, Treat), subjects were carefully instructed. In the scanner, participants laid supine, with their head resting in foam padding to reduce head movements. The noT and Treat versions of the paradigm were performed in two separate fMRI sessions. Session order and stimulus lists were counterbalanced across participants. Prior to each session, subjects had to complete a practice run (10 R, 10 U, 20 P) where additional visual feedback was given (“correct”, “wrong”, and “faster!”). As for suggestive treatment, the practice run was performed prior to the induction of hypnosis in order not to irritate participants subsequent to the task-specific suggestions. All participants performed close to ceiling in the practice run. The scanning session ended with acquisition of a T1-weighted structural image of the brain.

### MRI data acquisition

Functional images were acquired on a 3 Tesla magnetic resonance scanner (Magnetom Allegra, Siemens AG, Erlangen, Germany) in combination with a single channel transmit/receive head coil (RAPID Biomedical GmbH, Rimpar, Germany). During the experimental runs, a gradient-echo echo-planar pulse sequence (EPI) was applied to measure the T2*-weighted blood oxygen level dependent signal. The following parameters were used: repetition time = 2200 ms, echo time = 39 ms, flip angle = 90°, field of view = 230 mm, matrix size = 64 x 64, number of slices = 34, slice thickness = 3.0 mm, interslice gap = 0.6 mm, isotropic voxel size of 3.6 mm^3^. Ascending slice acquisition was parallel to a tangent plane touching the inferior surfaces of the orbitofrontal cortex and the cerebellum. Scan time per experimental run was 774 s, corresponding to 350 EPI volumes. To obtain a high resolution T1-weighted structural image for later coregistration purposes, a magnetization prepared rapid acquisition gradient echo sequence was employed (repetition time = 2080 ms, echo time = 3.93 ms, inversion time = 1100 ms, flip angle = 12°, field of view = 256 mm, matrix size: 256 x 256, voxel volume = 1 mm^3^, slice orientation: sagittal, scan time = 467 s).

### Data analysis

#### Behavioral data

Responses obtained from the lexical decision task were analyzed for reaction times (RT) and errors. For RT analysis only correct lexical decisions were included. Pseudoword trials were distractors of no interest and not further analyzed. Individual mean RT data for R and U trials were then fed into a 2 x 2 x 2 repeated measures analysis of variance (ANOVA) with factors Suggestibility (LS vs. HS), Treatment (noT vs. Treat), and Semantic Relatedness (R vs. U). A second ANOVA was performed on rates of erroneous responses calculated as the percentage of incorrect responses per trial type. Significance levels were set to a level of p < 0.050.

#### MRI data

Imaging data preprocessing and statistical analyses were performed using the software package SPM8 (Wellcome Department of Cognitive Neurology, London, UK). Preprocessing at the single subject level included slice time correction, spatial realignment, and coregistration of the T1 image to the EPI series. White and grey matter information yielded by segmentation of the T1 image was then subjected to the spatial normalization procedure realized by the DARTEL process stream [36]. During transformation into standard Montreal Neurological Institute (MNI) space, EPI series were resliced to a spatial resolution of isotropic 2 mm^3^ and smoothed using a Gaussian kernel with 8 mm full width at half maximum.

Preprocessed data were analyzed using a hierarchical standard modeling approach. At the single subject level, a session separated General Linear Model was set up with onsets for each condition of correct R, U, and P trials. As conditions of no interest, onsets of incorrect decisions were added to the design matrix as were the spatial realignment parameters. Resulting stick functions were convolved with the canonical hemodynamic response function and its time derivative. To remove low-frequency scanner drifts data were high-pass filtered with a frequency cutoff at 128 s, and an autoregression model of polynomial order 1 was used to account for temporally correlated residual errors.

Upon individual model estimation, we first tested for group differences between high- and low-suggestible subjects. Neural priming effects under normal wakefulness were computed for each subject by contrasting neural activities associated with semantically unrelated (U) minus semantically related (R) trials using the condition-specific contrast images. These contrast images were subjected to a two-sample t-test. The same procedure was repeated to test for possible group differences of neural priming effects under hypnotic suggestion.

For reasons explained in the Results section, in a second analysis fMRI data were pooled across all 24 participants to investigate possible treatment effects irrespective of individual suggestibility. Contrast images representing the main effects of R, and U against baseline for noT, and Treat were propagated to a random-effects analysis, implemented as flexible factorial design. This model comprised one within-subjects factor with four levels (R_noT_, U_noT_, R_Treat_, U_Treat_) by concatenating the two underlying factors. A second factor modeled subject-related variance. After model estimation, two one-sided t-contrasts [(U_noT_ - R_noT_) - (U_Treat_ - R_Treat_)], and [(U_Treat_ - R_Treat_) - (U_noT_ - R_noT_)] were computed to test for significant Treatment-by-Semantic Relatedness interactions.

Statistical parametric maps resulting from the analyses described above were thresholded at p < 0.005 at the voxel level in combination with a cluster extent threshold that required cluster sizes of contiguously significant voxels to survive a level of p < 0.050, corrected for multiple comparisons using the topographical false discovery rate (FDR) correction method [37].

Since the measure of suggestibility is a continuous variable, we also explored whether participants’ magnitude of treatment-induced neural priming modulation correlated with the individual HGSHS:A score. Contrast images representing the modulation of neural priming [(U_noT_ - R_noT_) - (U_Treat_ - R_Treat_)]) were computed at the single-subject level and correlated with the respective HGSHS:A scores in SPM8. Correlation analyses were only performed for voxels showing significantly less priming under Treat compared to noT. Significance was assessed at an uncorrected voxel height threshold of p < 0.050 combined with a cluster extent threshold of 10 contiguous voxels.

## Results

### Behavioral data

Averaged across participants, 29.8 R_noT_ trials, 28.7 U_noT_ trials, 29.5 R_Treat_ trials, and 27.9 U_Treat_ trials were correct, corresponding to an overall error rate of 3.4%. An ANOVA performed on mean reaction times of correct trials revealed significant semantic priming, i.e., faster responses to semantically related than to semantically unrelated targets (see Table 1; main effect of Semantic Relatedness, F(1, 22) = 4.54, p = 0.045). Overall, reaction times were slower after suggestive treatment than at normal wakefulness (F(1, 22) = 74.38, p < 0.001). However, reactions times were not significantly modulated by Suggestibility (F(1, 22) = 0.04, p = 0.843) and, moreover, there were not any significant interactions between factors Suggestibility and Semantic Relatedness, Treatment and Semantic Relatedness, or Suggestibility, Treatment and Semantic Relatedness (all F(1, 22) < 0.06, all p > 0.812).

**Table 1:** Behavioral results.

	R_noT_	U_noT_	R_Treat_	U_Treat_	Priming_noT_	Priming_Treat_
**Reaction times [ms]**						
**HS**	650 (27)	720 (29)	722 (61)	794 (62)	70	72
**LS**	641 (33)	720 (40)	700 (46)	778 (52)	79	78
**Error rates [%]**						
**HS**	0.5 (0.3)	4.3 (1.1)	1.7 (0.6)	6.2 (1.4)	3.8	4.5
**LS**	0.7 (0.4)	4.7 (1.7)	1.3 (0.7)	8.3 (1.4)	4.0	7.0

Mean reaction times and error rates associated with related (R), and unrelated (U) trials under normal wakefulness (noT), and after hypnotic-suggestive treatment (Treat) for high- (HS) and low-suggestible (LS) participants. The standard error of the mean (HS: n = 14, LS: n = 10) is given in parenthesis.

Priming was also found for error rates as dependent variable (see Table 1), indicated by a significant main effect of Semantic Relatedness (F(1, 22) = 40.88, p < 0.001). Furthermore, a significant main effect of Treatment was observed (F(1, 22) = 6.07, p = 0.022) reflecting higher error rates under hypnotic suggestion than at normal wakefulness. There were no other significant effects (all F(1, 22) < 1.96, all p > 0.175).

### fMRI data

**Fig 1:**
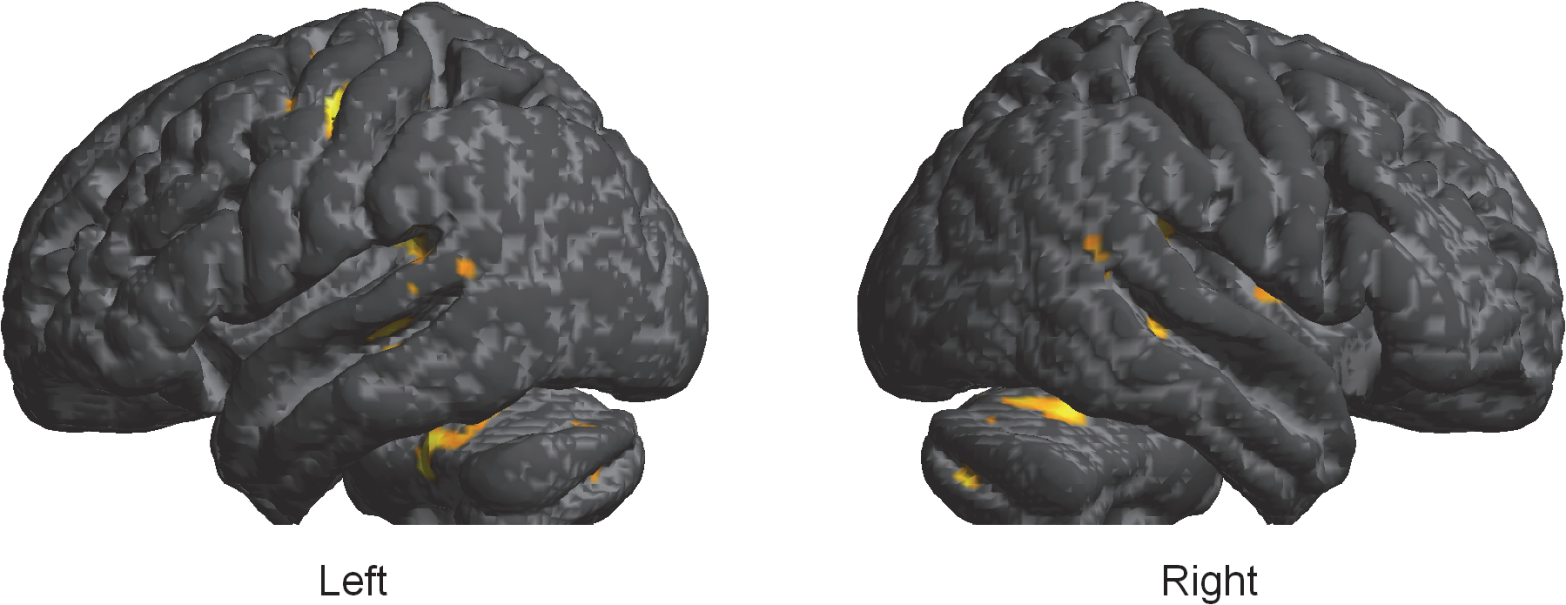
Effects of significantly attenuated neural semantic priming after hypnotic-suggestive treatment relative to normal wakefulness. The statistical parametric map reflecting the directed Treatment-by-Semantic Relatedness interaction t-contrast [(U_noT_ - R_noT_) - (U_Treat_ - R_Treat_)] was surface-rendered on the group averaged T1 image using SPM8.

**Fig 2:**
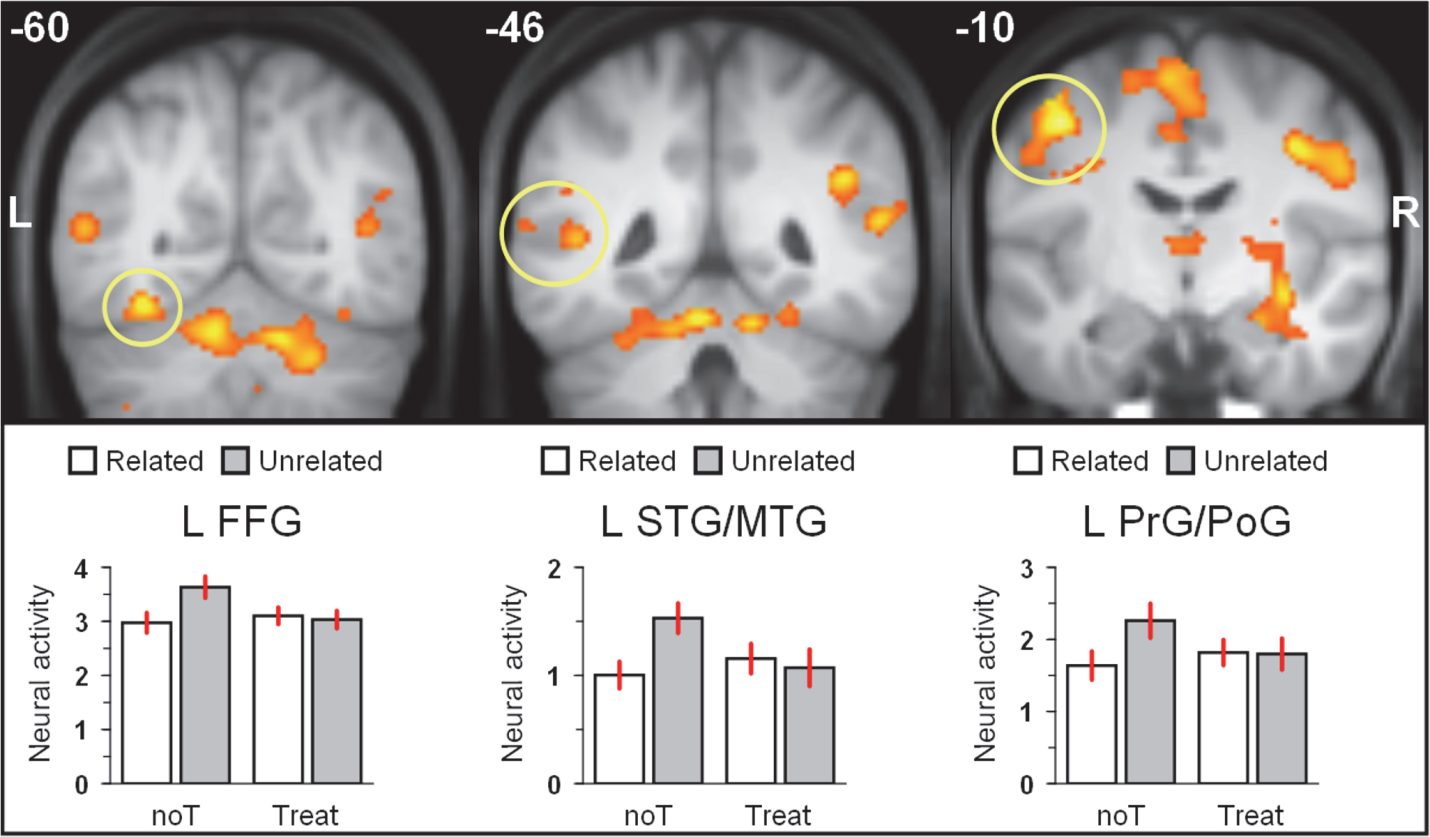
Selected coronal sections showing significantly attenuated neural semantic priming after hypnotic-suggestive treatment relative to normal wakefulness. The statistical parametric map associated with the contrast [(U_noT_ - R_noT_) - (U_Treat_ - R_Treat_)] was overlaid on the group averaged T1 image using MRIcron (http://www.mccauslandcenter.sc.edu/mricro/mricron/). Numbers in white color denote the y-coordinates of the slices in MNI space. From selected regions (yellow circles) the magnitude of neural activation associated with related, and unrelated trials at normal wakefulness (noT), and after treatment (Treat) was extracted and averaged. Error bars represent the standard error of the mean (24 participants). Abbreviations: L: left, R: right, FFG: fusiform gyrus, MTG: middle temporal gyrus, PoG: postcentral gyrus, PrG: precentral gyrus, STG: superior temporal gyrus.

The initial t-tests did not reveal any significant differences between high- and low-suggestible participants in the magnitude of semantic priming under normal wakefulness or treatment. Therefore, in a second analysis, data were collapsed across HS and LS participants to identify treatment effects on neural semantic priming irrespective of hypnotic suggestibility. Comparing both treatment conditions for significant differences, reduced neural priming after hypnotic-suggestive treatment relative to normal wakefulness was observed in the bilateral fusiform gyri (FFG; Brodmann area (BA) 19/37), and in the cerebellum (see Figs 1 and 2 and Table 2). Significant effects were also evident in the right superior (STG; BA 41) and middle (MTG; BA 21) temporal gyri extending into the putamen, the hippocampus, and the bilateral thalami. In the left hemisphere, there was also a posterior temporal cluster including parts of the left STG (BA 42) and the MTG (BA 21). Two further clusters encompassed portions of the bilateral precentral (PrG; BA 4/6) and postcentral (PoG; BA 3) gyri, and the SMA proper (BA 6). Parameter estimates depicted in Fig 2 suggest that priming was present at normal wakefulness and absent after hypnotic-suggestive treatment. The interaction contrast testing for significantly greater priming after hypnotic suggestion than at normal wakefulness did not reveal any significant results. For comparison purposes, significant effects of priming at normal wakefulness as well as the average priming effect over both treatment conditions are provided as Supporting Information (see S1 Fig, S1 Table and S2 Table).

**Table 2:** Brain regions demonstrating effects of significantly attenuated priming after hypnotic-suggestive treatment compared to normal wakefulness, tested by the contrast [(U_noT_ - R_noT_) - (U_Treat_ - R_Treat_)].

	Region	BA	Cluster size	Peak voxel (MNI space)			
				x	y	z	z-score
R	Cerebellum	-	2801	12	-66	-26	4.89
L	Cerebellum	-		-14	-52	-24	4.18
L	Fusiform gyrus	37		-32	-60	-14	3.94
R	Fusiform gyrus	19		38	-66	-16	3.03
R	Thalamus	-	1799	14	-28	2	4.58
R	Superior temporal gyrus	41		40	-34	24	3.99
R	Putamen	-		34	-6	-10	3.95
L	Thalamus	-		-12	-24	8	3.80
R	Hippocampus	-		26	-6	-20	3.56
R	Middle temporal gyrus	21		46	-32	-2	3.08
L	Precentral gyrus	6	1604	-42	-10	48	4.35
L	Supplementary motor area proper	6		-6	-12	62	3.52
L	Postcentral gyrus	3		-52	-16	32	3.26
R	Supplementary motor area proper	6		12	-2	48	3.29
R	Precentral gyrus	4	616	36	-12	40	4.10
R	Postcentral gyrus	3		52	-14	36	3.93
L	Middle temporal gyrus	21	832	-46	-42	6	3.46
L	Superior temporal gyrus	42		-56	-38	12	3.40

Cluster size refers to number of voxels.

Abbreviations: L: left, R: right, BA: Brodmann area, MNI: Montreal Neurological Institute.

There was no significant correlation between the magnitude of treatment-induced modulation of neural priming and participants' HGSHS:A score. We only found three small clusters that did not, however, pass the initially defined extent threshold of 10 contiguous voxels, and therefore failed significance: 7 voxels in the right STG, 1 voxel in the right MTG, and 7 voxels in the right hippocampus.

## Discussion

In the present fMRI study we compared behavioral and neural semantic priming effects under normal wakefulness and after hypnotic suggestion aimed at reducing semantic processing of the primes. We assumed that our hypnotic suggestion should attenuate semantic processing of the primes by means of establishing an attentional set that discourages word reading similar to the studies using the induction task paradigm in combination with masked primes [4–7]. Based on the brain activation pattern modulated by hypnotic suggestion, we were able to determine whether suggestion reduces automatic and/or strategic semantic processes. If hypnotic suggestion influences automatic semantic processing, priming should be affected in ventral occipito-temporal cortex such as the fusiform gyrus (FFG), a brain region that has previously been associated with automatic semantic processing [5, 27, 38–40]. If the treatment modulates strategic semantic processing, reductions in priming can be expected to emerge in the superior temporal gyrus (STG), a region implicated in strategic semantic processing (e.g., [27, 41]).

Inspection of Fig 2 shows that the activation level associated with U trials under noT more strongly differed from the other conditions, which more or less showed the same level. Irrespective of the precise mechanisms in question, which can account for this result pattern, it clearly demonstrates significant priming, defined as difference between unrelated and related trials, only under noT, whereas treatment completely abolished priming. However, as the neutral activation level in the lexical decision task at normal wakefulness is not known in the present study, it is difficult to infer from the data of the present study alone, how factors Semantic Relatedness and Treatment elicited this interaction pattern. Two interpretations based on automatic vs. strategic priming mechanisms are conceivable: (i) Hypnotic suggestion to treat prime words as meaningless letter strings could have generally reduced activity in semantic brain areas by decreasing their sensitivity to process semantic information compared with noT [5, 6]. The related condition under noT could then have reduced the activity compared with the corresponding unrelated condition by preactivation of semantic features. Hence, the priming effect under noT would reflect facilitation of semantically related targets by automatic semantic activation [1]. (ii) Hypnotic suggestion could have diminished the application of controlled semantic processing strategies such as semantic matching [24]. Unlike expectancy generation, semantic matching can operate even at relatively short prime target stimulus onset asynchronies of 200 ms [42], as in the present study. Semantic matching strategies, only applied under noT, would enhance the activity level of the unrelated condition compared with the corresponding related condition because the failure to detect a semantic relation between prime and target induces elaborated post-lexical integration processes. According to this interpretation, the priming effect under noT would reflect the increased semantic matching costs of semantically unrelated targets.

As we will discuss in more detail below, both suggested mechanisms are not mutually exclusive, but could have conjointly influenced priming by modulating activity in different semantic areas. Semantic activation could have driven modulation of priming effects in the FFG, an area previously shown to be sensitive to automatic semantic processing. In contrast, modulation of priming in the STG, the pre- and postcentral gyri (PrG/PoG), and the supplementary motor area proper (SMA proper) could reflect semantic matching strategies, in line with an involvement of these areas in strategic semantic processing [27]. Thus, our neuroimaging data indicate that hypnotic suggestion modulates both automatic and strategic aspects of semantic processing.

Hypnotic suggestion similarly affected neural priming in high- and low-suggestible participants. Hence, our suggestion seems to have directed participants’ attention away from the meaning of the prime words irrespective of the depth of the trance state as also observed in an earlier study with the Stroop task [13]. It is also possible that our measure of hypnotic suggestibility obtained in a quiet situation was not a valid index of hypnotic suggestibility in a scanning environment (i.e., placement within the narrow magnet, gradient noise) due to interindividual differences in distractibility rendering our group division into high- and low-suggestible individuals less valid. Although previous research has shown that the scanning environment does not affect the ability to respond to suggestions under hypnosis [43], the scanning environment may individually affect the deepness of the trance state [28], which is not captured by measures of suggestibility obtained outside the scanner. In addition, sample sizes of both subgroups were quite low, which decreases statistical power to detect reliable effects. This is a major limitation of the present study.

In contrast to our expectations, hypnotic suggestion only modulated neural semantic priming but not behavioral priming. The lack of treatment effects on behavioral priming could be accounted for by the general effect of hypnotic suggestion on participants’ ability to perform the lexical decision task. Hypnotic suggestion not only prolonged reaction times and increased error rates but also led to a considerably higher variability compared with normal wakefulness (see Table 1). This indicates that suggestive treatment impaired participants to perform the lexical decision and increased the noise in the behavioral data. Hence, differences in priming between normal wakefulness and the suggestion condition might be masked by heightened response variability. That increased jitter might be due to the complex hypnotic suggestion that prompted participants in all trials to construe the prime as meaningless symbol on the one hand, but to subsequently discriminate between a meaningful word and a pseudoword upon presentation of the lexical decision target on the other hand. Brain activation as a measure of priming might be less affected by response variability because neuroimaging methods are able to directly capture activity in semantic brain areas without influence of possibly compromised subsequent decision or motor response stages. For that reason, dissociations between behavioral and neurophysiological measures of priming similar to the present one have been observed in several earlier studies (e.g., [44, 45]; see also [46], for a discussion).

Significant treatment differences due to reductions in priming after suggestion were found in an FFG region close to the occipito-temporal clusters found in our previous studies [5, 27]. The FFG has been implicated in various aspects of semantic processing including the retrieval of visual features [47]. As FFG activation has been frequently observed under conditions emphasizing automatic processing [40] including masked semantic priming [5, 27], it likely indexes a modulation of automatic semantic processes. The suggestive treatment-induced attenuation of priming in a brain region implicated in automatic semantic processing is in line with recent behavioral, electrophysiological, and neuroimaging findings showing that the magnitude of automatic semantic priming elicited by unconsciously perceived masked primes depends on the specific task set active prior to performing the lexical decision task [4–7]. In those studies, unconscious automatic semantic priming was boosted when the lexical decision task was preceded by an “induction task” (e.g., semantic categorization) that led subjects to direct their attention to semantic features. Conversely, semantic priming was diminished when the induction task was non-semantic in nature (e.g., perceptual discrimination). In line with the present results, we recently observed in an fMRI study an induction task-induced modulation of masked priming in the FFG [5]. These findings were explained by assuming that task sets sensitize task-relevant and de-sensitize task-irrelevant processing pathways by top-down attentional control (“attentional sensitization” framework [6]). Comparable to the modulation through tasks sets, attempts have also been made to alter cognitive processing by administering hypnotic suggestions (e.g., [48, 49]). Raz and colleagues have shown that it is possible to reduce or even eliminate the Stroop effect by administering task-specific posthypnotic suggestions [12–15, 28].

However, clusters with Treatment-by-Semantic Relatedness interactions also encompassed portions of the STG, the middle temporal gyrus (MTG), the PrG and PoG, and the SMA proper. These regions coincide with those that have previously demonstrated greater priming under unmasked than masked priming conditions in our previous study [27]. For the areas of overlap (STG, PrG, PoG, and SMA proper) we have previously suggested that they may mediate strategic processes as opposed to automatic processes associated with semantic memory representation [27]. Given the short stimulus onset asynchrony of 200 ms, the strategic process most likely applied by participants is semantic matching [18, 24–26, 50], a strategy holding that subjects use the degree of semantic relatedness between prime and target to aid the lexical decision process. In case both meanings match (R trials), the lexical decision is then biased towards a “word” response whereas the lack of a semantic relationship might bias a “pseudoword” response, thus facilitating decisions on R in comparison to U targets.

Other regions which showed a treatment-induced reduction of semantic priming were the cerebellum and the hippocampus. We consider activation in those areas as not specific to semantic processing because they presumably play a more general role in language and memory. The cerebellum is involved in a variety of motor and cognitive tasks (see [51], for a review), including semantic memory [52–54], making it difficult to delineate the cerebellum’s specific contributions to semantic priming, may they be related to motor or cognitive operations. The hippocampus is a crucial structure for episodic memory [55] although its activity has been sometimes found to be modulated in semantic tasks including priming [27, 56]. The hippocampus’ greater activation during U trials might reflect its implicit attempt to establish a link between the prime and the target, given that there is no pre-existing association. During R trials, in contrast, the per se existing association between prime and target may have required less learning-related activation. We therefore assume priming effects in the hippocampus have arisen from semantic matching strategies. This assumption is supported by the observation that priming effects in the hippocampus were restricted to unmasked priming and not present during masked priming in our earlier study [27].

Given the known role of the brain regions, which showed a treatment-induced reduction in neural priming, for semantic processing, we propose that suggestions have targeted both automatic and strategic semantic processing, conjointly reducing the magnitude of neural semantic priming.

A limitation of the present study is the lack of post-scanning measures assessing participants’ hypnotic state directly after the Treat condition. Furthermore, the precise cognitive and neural mechanisms underlying the observed treatment-induced attenuation of brain activation cannot be unequivocally determined based on the present study alone. As outlined above, the reported treatment effects might have emerged because the hypnotic suggestions specifically targeted automatic and strategic prime processing by reducing the sensitivity of semantic pathways to analyze the meaning of the prime. This interpretation is in line with findings of a reduction of unconscious semantic priming at a neural [5, 6] or behavioral level [4, 6] when task sets direct attention away from semantic to non-semantic perceptual stimulus features. Similar effects on visible semantic priming have been observed in studies varying the task participants had to perform on the prime [57]. The present study confirms and advances this earlier evidence by showing that such a modulation of semantic priming can also be achieved by giving a verbal instruction via hypnotic suggestion to process the prime non-semantically. However, we cannot entirely rule out that the treatment-related attenuation of semantic priming arose from a more general reduction of attention to the prime words, dampening priming-related brain activation. In this regard, it would be interesting for future research to compare effects of hypnotic suggestions identical or similar to those used in the present study with effects evoked by simply instructing participants to “pay less attention to the primes”.

## Conclusions

We investigated modulation of supraliminal semantic priming effects through hypnotic suggestions aimed at dampening semantic processing of the prime. Suggestion significantly attenuated priming effects in several brain regions including portions of the fusiform gyrus, the superior and middle temporal gyri, the pre- and postcentral gyri, and the supplementary motor area proper. Based on previous findings (e.g., [27]), both automatic and strategic processes (i.e., semantic matching) were presumably affected by hypnotic suggestion. Hence, hypnotic suggestions seem to exert a global influence on language processing by establishing attentional sets that modulate semantic processing at an automatic and strategic level. Future studies should investigate whether it is possible to alter semantic priming through suggestions without prior administration of hypnosis.

## Supporting Information

S1 Fig Comparison of priming effects. Top panel: Priming at normal wakefulness [U_noT_ - R_noT_]. Middle panel: Average priming over conditions normal wakefulness and hypnotic suggestion [U_noT_ - R_noT_ + U_Treat_ - R_Treat_]. Lower panel: Hypnotic suggestion-induced modulation of priming [(U_noT_ - R_noT_) - (U_Treat_ - R_Treat_)]. The respective statistical parametric maps, thresholded at p < 0.005 (voxel level) and p < 0.05 (cluster level, FDR-corrected), were surface-rendered on the group averaged T1 image using SPM8.
(TIF)

S1 Table Peak coordinates in MNI space of brain regions showing significant semantic priming under normal wakefulness, tested by the contrast [U_noT_ - R_noT_]. (DOC)

S2 Table Peak coordinates in MNI space of brain regions demonstrating a significant average priming effect over conditions normal wakefulness and hypnotic suggestion [U_noT_ - R_noT_ + U_Treat_ - R_Treat_]. (DOC)
