# Supplementary figures and images for: Suggestion-Induced Modulation of Semantic Priming during Functional Magnetic Resonance Imaging
Source: PLoS One. 2015 Apr 29;10(4):e0123686. doi: 10.1371/journal.pone.0123686 (PMC4414585; doi:10.1371/journal.pone.0123686)

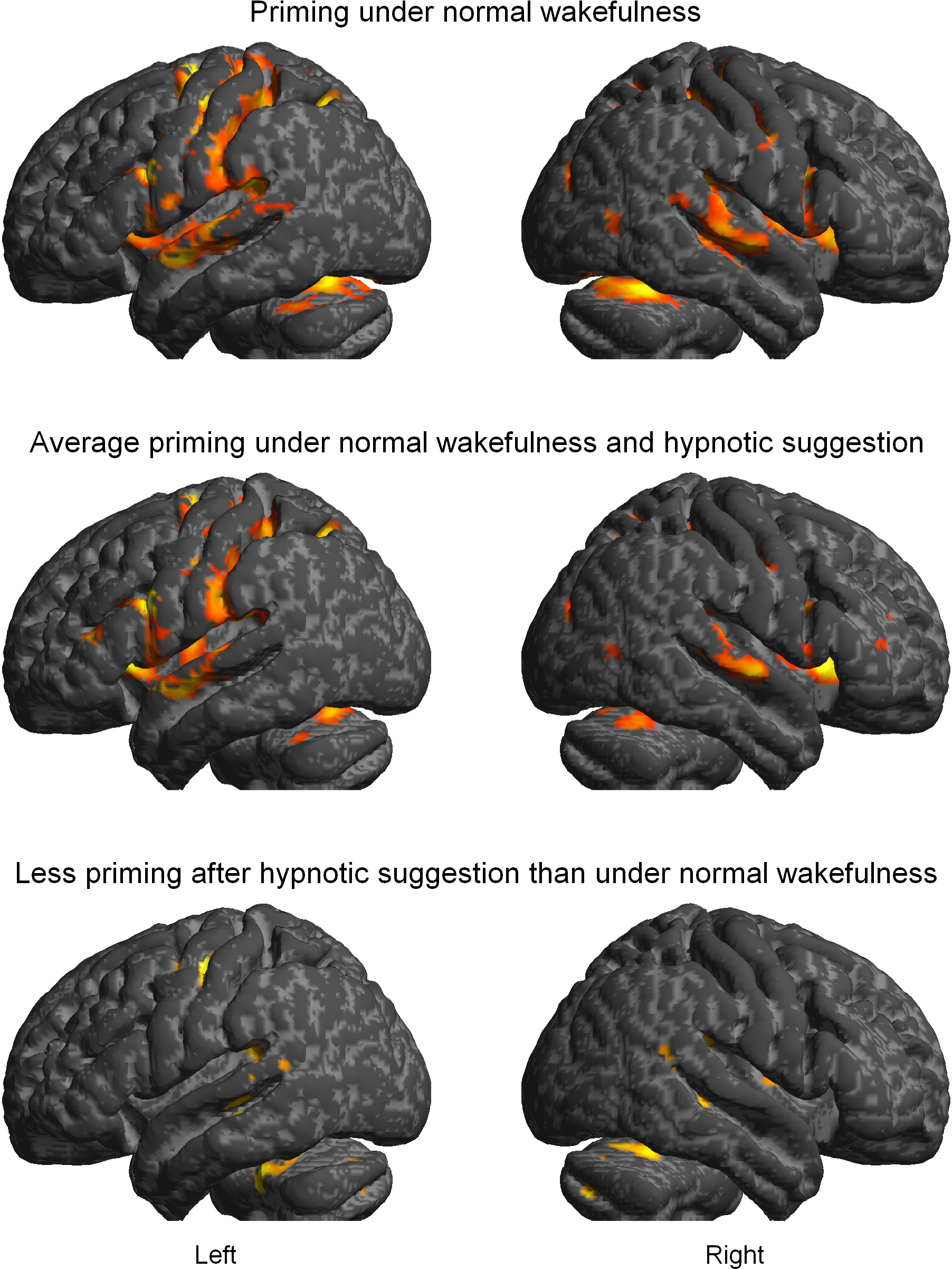

Supplement: S1 Fig — Top panel: Priming at normal wakefulness [UnoT - RnoT]. Middle panel: Average priming over conditions normal wakefulness and hypnotic suggestion [UnoT - RnoT + UTreat - RTreat]. Lower panel: Hypnotic suggestion-induced modulation of priming [(UnoT - RnoT) - (UTreat - RTreat)]. The respective statistical parametric maps, thresholded at p < 0.005 (voxel level) and p < 0.05 (cluster level, FDR-corrected), were surface-rendered on the group averaged T1 image using SPM8. (TIF) [file pone.0123686.s001.tif]
