# Supplementary material for: Suggestion-Induced Modulation of Semantic Priming during Functional Magnetic Resonance Imaging
Source: PLoS One. 2015 Apr 29;10(4):e0123686. doi: 10.1371/journal.pone.0123686 (PMC4414585; doi:10.1371/journal.pone.0123686)
Supplement: S1 Table — (DOC) [file pone.0123686.s002.doc]

**Table S1.** Peak coordinates in MNI space of brain regions showing significant semantic priming under normal wakefulness, tested by the contrast [U_noT_ - R_noT_].

|  | Brain region | BA | x | y | z | z-score |
| --- | --- | --- | --- | --- | --- | --- |
| L | Cerebellum | - | -16 | -62 | -20 | 4.44 |
| L | Fusiform gyrus | 19 | -34 | -62 | -14 | 4.45 |
| L | Fusiform gyrus | 37 | -38 | -48 | -16 | 3.73 |
| L | Hippocampus | - | -30 | -16 | -14 | 5.37 |
| L | Inferior frontal gyrus opercular part | 44 | -50 | 8 | 6 | 4.42 |
| L | Inferior temporal gyrus | 37 | -44 | -60 | -4 | 4.37 |
| L | Lingual gyrus | 18 | -16 | -84 | -6 | 4.56 |
| L | Lingual gyrus | 19 | -18 | -64 | -2 | 4.06 |
| L | Middle cingulate gyrus | 24 | -8 | 8 | 32 | 4.58 |
| L | Middle temporal gyrus | 21 | -46 | -46 | 10 | 3.92 |
| L | Postcentral gyrus | 3 | -52 | -18 | 44 | 4.65 |
| L | Postcentral gyrus | 3 | -54 | -18 | 32 | 4.56 |
| L | Precentral gyrus | 6 | -48 | -6 | 42 | 5.53 |
| L | Precentral gyrus | 6 | -54 | 6 | 30 | 5.28 |
| L | Precentral gyrus | 6 | -26 | -26 | 60 | 5.04 |
| L | Pre-supplementary motor area | 6 | -8 | 4 | 50 | 4.78 |
| L | Putamen | - | -20 | 18 | 2 | 5.06 |
| L | Superior parietal lobule | 7 | -18 | -64 | 54 | 4.32 |
| L | Superior temporal gyrus | 22 | -54 | -20 | 2 | 4.84 |
| L | Superior temporal gyrus | 42 | -52 | -34 | 18 | 4.63 |
| L | Superior temporal gyrus | 21 | -54 | 0 | -8 | 4.37 |
| L | Supplementary motor area | 6 | -2 | -6 | 56 | 4.55 |
| L | Thalamus | - | -12 | -20 | 4 | 5.01 |
| R | Anterior insula | - | 28 | 24 | -2 | 3.90 |
| R | Cerebellum | - | 12 | -72 | -40 | 4.66 |
| R | Cerebellum | - | 26 | -54 | -20 | 4.45 |
| R | Cerebellum | - | 14 | -46 | -18 | 4.40 |

**Table S1 continued.**

|  | Brain region | BA | x | y | z | z-score |
| --- | --- | --- | --- | --- | --- | --- |
| R | Fusiform gyrus | 19 | 24 | -60 | -16 | 4.03 |
| R | Fusiform gyrus | 37 | 26 | -52 | -18 | 3.97 |
| R | Hippocampus | - | 30 | -4 | -24 | 3.82 |
| R | Inferior frontal gyrus triangular part | 45 | 36 | 26 | 12 | 4.17 |
| R | Inferior temporal gyrus | 37 | 44 | -60 | -8 | 4.16 |
| R | Lingual gyrus | 18 | 22 | -84 | -6 | 4.57 |
| R | Middle temporal gyrus | 37 | 42 | -68 | 10 | 5.01 |
| R | Middle temporal gyrus | 22 | 62 | -14 | -8 | 4.10 |
| R | Postcentral gyrus | 3 | 52 | -14 | 36 | 4.59 |
| R | Postcentral gyrus | 3 | 26 | -24 | 60 | 4.54 |
| R | Precentral gyrus | 4 | 48 | -6 | 34 | 4.03 |
| R | Pre-supplementary motor area | 6 | 2 | 16 | 48 | 4.38 |
| R | Putamen | - | 30 | -14 | -8 | 5.72 |
| R | Superior parietal lobule | 7 | 20 | -60 | 56 | 3.81 |
| R | Superior temporal gyrus | 22 | 60 | -26 | 8 | 3.93 |
| R | Superior temporal gyrus | 41 | 48 | -34 | 14 | 3.65 |
| R | Supplementary motor area | 6 | 6 | -4 | 52 | 4.52 |
| R | Thalamus | - | 16 | -14 | 8 | 4.66 |

Abbreviations: L: left, R: right, BA: Brodmann area, MNI: Montreal Neurological Institute.
