# Supplementary material for: Suggestion-Induced Modulation of Semantic Priming during Functional Magnetic Resonance Imaging
Source: PLoS One. 2015 Apr 29;10(4):e0123686. doi: 10.1371/journal.pone.0123686 (PMC4414585; doi:10.1371/journal.pone.0123686)
Supplement: S2 Table — (DOC) [file pone.0123686.s003.doc]

**Table S2.** Peak coordinates in MNI space of brain regions demonstrating a significant average priming effect over conditions normal wakefulness and hypnotic suggestion [U_noT_ - R_noT_ + U_Treat_ - R_Treat_].

|  | Brain region | BA | x | y | z | z-score |
| --- | --- | --- | --- | --- | --- | --- |
| L | Cerebellum | - | -18 | -64 | -18 | 3.85 |
| L | Fusiform gyrus | 37 | -40 | -64 | -12 | 3.71 |
| L | Hippocampus | - | -30 | -14 | -16 | 4.78 |
| L | Inferior frontal gyrus opercular part | 44 | -48 | 8 | 6 | 4.39 |
| L | Inferior frontal gyrus triangular part | 45 | -48 | 30 | 12 | 3.99 |
| L | Inferior occipital gyrus | 37 | -42 | -62 | -6 | 5.02 |
| L | Inferior temporal gyrus | 37 | -40 | -46 | -14 | 3.90 |
| L | Insula | - | -32 | 18 | 2 | 4.32 |
| L | Lingual gyrus | 18 | -16 | -84 | -6 | 4.85 |
| L | Lingual gyrus | 18 | -14 | -66 | 0 | 3.90 |
| L | Middle cingulate gyrus | 32 | -6 | 12 | 42 | 4.20 |
| L | Middle occipital gyrus | 18 | -30 | -88 | 14 | 4.08 |
| L | Middle temporal gyrus | 22 | -60 | -8 | -8 | 4.30 |
| L | Postcentral gyrus | 3 | -58 | -18 | 30 | 3.96 |
| L | Postcentral gyrus | 3 | -52 | -20 | 46 | 3.93 |
| L | Precentral gyrus | 6 | -54 | 6 | 28 | 5.22 |
| L | Precentral gyrus | 6 | -48 | -6 | 42 | 5.06 |
| L | Pre-supplementary motor area | 6 | -10 | 4 | 50 | 5.75 |
| L | Putamen | - | -20 | 18 | 2 | 4.58 |
| L | Superior parietal lobule | 7 | -28 | -54 | 52 | 4.50 |
| L | Superior temporal gyrus | 22 | -54 | -18 | 2 | 4.73 |
| L | Superior temporal gyrus | 21 | -54 | -2 | -10 | 4.12 |
| L | Supplementary motor area | 6 | -14 | -6 | 60 | 4.07 |
| L | Thalamus | - | -10 | -18 | 4 | 4.74 |
| R | Cerebellum | - | 26 | -58 | -20 | 3.63 |
| R | Fusiform gyrus | 19 | 24 | -60 | -16 | 3.40 |
| R | Inferior frontal gyrus opercular part | 44 | 48 | 6 | 26 | 4.19 |

**Table S2 continued.**

|  | Brain region | BA | x | y | z | z-score |
| --- | --- | --- | --- | --- | --- | --- |
| R | Inferior frontal gyrus triangular part | 45 | 46 | 36 | 8 | 3.40 |
| R | Inferior temporal gyrus | 37 | 42 | -56 | -6 | 3.97 |
| R | Insula | - | 32 | 22 | 10 | 4.87 |
| R | Lingual gyrus | 18 | 22 | -82 | -6 | 4.65 |
| R | Lingual gyrus | 17 | 8 | -76 | 0 | 3.89 |
| R | Middle temporal gyrus | 37 | 44 | -68 | 8 | 3.87 |
| R | Postcentral gyrus | 3 | 52 | -14 | 38 | 3.10 |
| R | Precentral gyrus | 6 | 56 | 4 | 40 | 3.53 |
| R | Pre-supplementary motor area | 6 | 2 | 16 | 46 | 4.40 |
| R | Putamen | - | 30 | -14 | -8 | 5.10 |
| R | Superior parietal lobule | 7 | 18 | -62 | 56 | 3.83 |
| R | Superior temporal gyrus | 21 | 66 | -10 | -2 | 4.08 |
| R | Thalamus | - | 14 | -14 | 6 | 4.34 |

Abbreviations: L: left, R: right, BA: Brodmann area, MNI: Montreal Neurological Institute.
